# The impact of internet use on health status among older adults in China: The mediating role of social support

**DOI:** 10.3389/fpubh.2023.1108096

**Published:** 2023-02-22

**Authors:** Yiting E, Jianke Yang, Long Niu, Chunli Lu

**Affiliations:** ^1^Department of Sociology, School of Humanities and Social Sciences, Xi'an Jiaotong University, Xi'an, China; ^2^School of Normal Education, Longyan University, Longyan, Fujian, China

**Keywords:** internet use, social support, health status, older adults, mediation analysis

## Abstract

**Background:**

With the popularization of the Internet, the use of the Internet is becoming more and more important in the daily life of older adults. However, previous research mainly focuses on Internet use and health in general, and the mechanism of this effect remains to be studied. To bridge this gap, this study aims to explore the mediational effects of social support between Internet use and health among older adults in China.

**Methods:**

The data used in this article are from the 2021 Chinese General Social Survey (CGSS). Social support is divided into two aspects and four dimensions: informal social support (relatives support, friends support, neighbors support) and formal social support (social insurance). This article uses the nested multivariate OLS regression models to analyze the impact of Internet use on health. Furthermore, Finally, SPSS macro PROCESS is applied to test their mediation effects.

**Results:**

Informal social support positively influenced the health status among older adults, while formal social support did not. Among the three types of informal social support, relatives support and friends support significantly affected health status among Chinese older adults. Regarding social support differences between urban and rural areas, it was found that relatives support is a positively significant factor for rural older adults, while friends support is significant for urban older adults.

**Conclusions:**

Since Internet use has many ways of impacting health status, social support only plays a partial mediating role in this study. It recommends that the government should take compelling measures to encourage and promote the use of the Internet among older adults and obtain various social support to improve their health status.

## Introduction

China's population has been rapidly aging over the last few decades. By the end of 2020, China had 264 million people over the age of 60, accounting for 18.9% of the total population; this figure is expected to rise to more than 300 million by the end of 2025 (1). The World Health Organization (WHO) has defined active aging as “the process of optimizing the opportunities for health, participation, and safety to improve the quality of life as the person ages” (2). Consequently, improving the health of older adults has emerged as a priority for health policymakers. On the other hand, the arrival of the Internet era has greatly changed the lives of older adults. According to the 45th Statistical Report on China's Internet Development, the number of Internet users in China had reached 904 million by 2020, with 6.7% being over the age of 60 (3). Especially during the COVID-19 pandemic, Internet use has become a trend. Compared to the pre-lockdown period, Internet usage among older adults has increased significantly (4). By December 2021, the Internet penetration rate of the older adults reached 43.2% (5). Therefore, with China's aging population, widespread Internet use has emerged as a new factor that may have an impact on their health status.

Researchers have recently begun to focus on the relationship between Internet use and the health of older adults. Studies have shown that older adults can not only search for health information through the Internet, but also help them build new social networks and reduce social isolation (6, 7). For example, Bretherton conducted a survey of 214 older Australians and found that higher levels of social support mean not using mental health services (8). Researchers have also investigated the role of social support on the health of older adults, and their findings showed that social support has a direct or indirect impact on the health of older adults (9, 10). For example, a prospective repeated measurement cohort study on participants aged 60 and over showed that the higher the social support, the lower the psychological distress (11).

While previous studies have demonstrated that Internet use is associated with health status, few studies have explored the impact mechanism and path of Internet use on health status among older adults. Social support may be a very important mediating factor in this process. Internet use may not only have a direct effect on the health of older adults, but also have an indirect effect on the health of older adults through the mediating variables of social support. However, many previous studies have focused on the direct effects of social support on the physical and mental health of older adults, little is known about their underlying mechanisms. Furthermore, the correlation between social support and personal health has been analyzed in existing studies (12), and Internet use may have effects on social support (13–15). Therefore, whether and how social support mediates the relationship between Internet use and older adults' health status remains to be further tested.

On this basis, this study aims to study the impact of Internet use on the health status of older adults in China, focusing on the mediating role of social support in this process. By using a nationally representative survey and multiple mediating models, this study empirically tests whether different types of social support are potential mediation mechanisms for the relationship between Internet use and health status. In addition, we try to discover whether such impact mechanisms differ significantly between rural and urban areas based on the Chinese context to enrich the existing studies.

## Literature review and research hypotheses

### Internet use and health

At present, the Internet is now widely used around the world and has become a way of life for an increasing number of people. Scholars have conducted numerous empirical studies on the impact of Internet use on health outcomes. On the one hand, Internet use by older adults has an impact on their physical health. The Internet provides a platform for people to communicate and help them acquire more health knowledge, which is of great significance for improving their health (16–19). For example, Flynn Kathryn and his colleagues pointed out in the study that one-third of the respondents searched their health or medical care information on the Internet (20). In terms of disease intervention, Internet interventions have been widely adopted to aid disease management in a variety of areas, such as HIV and AIDS, malaria, tuberculosis, diabetes, asthma, obesity, and smoking (21), thus reducing mortality (22). Besides, some scholars' research showed that the use of smartphones can effectively improve the elderly's self-health evaluation (23). On the other hand, it is also closely related to their mental health. As it is known to all, with the growth of age, the social interaction of the elderly gradually shrinks, and the sense of loneliness of the elderly increases significantly. It is precisely because the Internet has brought about an increase in social interaction, which can effectively reduce the loneliness of the elderly, have a positive impact on their emotions, enable older adults to obtain a better psychological state, and reduce their mental diseases, such as depression, anxiety, post-traumatic stress disorder (PTSD) and stress (24), so older adults can use the Internet to expand social interaction, reduce loneliness, and improve mental health (25, 26). For example, Atsushi found that when compared with the elderly who do not use the Internet at all, the elderly using the Internet every day can keep close contact with society, meet friends more frequently, and effectively improve their subjective well-being (27); Some researchers have also revealed the relationship between Internet use and subjective well-being of older adults through path analysis, and their findings suggest that the use of the Internet can enhance the ability of older adults to maintain close intergenerational relationships and thus contribute to their subjective well-being (28). In short, although considerable studies have confirmed the effects of internet use on older adults' heath and explained them from the perspectives of social relationships and lifestyles, there are still some gaps in existing research. First, since most of the above content comes from western culture, it is necessary to identify this phenomenon in the Chinese context to understand the differences between these empirical studies and those reported in the previous literature. Second, the direct effect of internet use on older adults' health has been well confirmed, while the possible mediating mechanism is not clear.

### Social support and health

In the early 1970s, social support was formally put forward as an academic concept and a professional term. Scholars in various research fields put forward different definitions of social support, and there are two main perspectives. First, in the perspective of social interaction, social support is not only one-way care and help, but also a social exchange and a social interaction between people in most situations (29, 30). Bernard believes that social support is a collection of families, friends, and social institutions that people rely on to meet their social, physical, and psychological needs (31). Second, in the perspective of social resources, social support refers to a kind of behavior or information that individuals feel is concerned, respected, and valued by the members of their social network, and also comes from the help of social relations and the resource exchange of members in the social network (32). House and Turner demonstrated that there were meaningful groups around individuals, such as family members, friends, colleagues, relatives, and neighbors, who had positive support and effects on individuals, including practical help, social emotional help, and information help (33, 34). Cullen defines social support as a variety of material and spiritual information that can be received by individuals from communities and social networks (35). In general, social support refers to the process by which individuals receive and use help from the government, social organizations, and others (36).

Social support can be divided into formal social support and informal social support. Formal social support is defined as the support provided by the government, institutions, communities, and other formal organizations for vulnerable groups, such as endowment insurance and the system of medical safety and security (37). In recent decades, China's social security has made phenomenal progress, with the widespread establishment and dramatic expansion of the social insurance system. Since 2003, China has established a basic social insurance system that covers almost all rural and urban residents till now, which is divided into two parts: health insurance and endowment insurance. For health insurance, the three pillars of the system are the New Rural Cooperative Medical Scheme (NCMS), Urban Resident Basic Medical Insurance (URBMI), and Urban Employees' Basic Medical Insurance (UEBMI); For endowment insurance, the new rural endowment insurance and the urban endowment insurance were included in the basic endowment insurance for urban and rural residents (38). In our study, we used Zhang and Shen's research framework, that is, social insurance as a key indicator for measuring formal social support (39, 40). Unlike formal support from official organizations, in Berkman and Cantors' research, informal social support mainly refers to emotional, behavioral support, which is provided by family members, neighbors, friends, and colleagues (41, 42). For older adults, social support is a known contributor to health in the general (43, 44). As they withdraw from the main areas of social life, social interaction is greatly reduced, which will lead to their mental health problems such as loneliness and depression. At this time, social support and its network members can help the elderly actively seek help, and can also serve as internal support to promote their mental health (45). For example, Adams found in his research that the elderly living alone may rely on friends and neighbors to establish similar relationships with their families (46). Using a sample survey from Chengdu, Sichuan Province, China, Tang et al. found that informal social support had significant positive health effects, especially on those with high age and agricultural household registration (47). However, studies have not examined the independent effects of the two types of social support on the health of older adults separately. In the following sections, we elaborate on these two lines of research.

### Internet use, social support, and health

Recently, with the widespread use of the Internet, scholars began to focus on the relationship between Internet use, social support, and health. On the one hand, there was a significant correlation between specific Internet use (including online chat, game, and entertainment use) and social support (48, 49). For example, a study in Finland found that as a source of social support, the Internet can help people obtain emotional and informational support (50). According to a study based on seven European countries, researchers believe that the use of the Internet is more closely related to social support and subjective health than the use of other media (51). Meanwhile, other scholars further pointed out that the main purpose of people accessing the Internet is to maintain the existing social support network in real life, rather than to develop online virtual social networks (52). Moreover, research on older adults in China also found that the main purpose of older adults use the Internet is to keep in touch with relatives and friends, which enables them to expand or maintain social contacts and increase access to information. A high level of Internet use means the elderly have more opportunities to interact and connect with relatives, friends, and society, thus promoting social support (53). On the other hand, many scholars further pointed out the relationship between Internet use, informal social support, and health. Kang pointed out that online chat can improve social support and reduce depression (54). Some scholars found in the survey that the use of the Internet can reduce the loneliness of elderly Polish males, increase social support, and have a better quality of life (55).

To conclude, although scholars recently have started to examine the relationship between Internet use, social support, and health, they have not used the same research framework, and have only considered the relationship between the two of them. Therefore, this study will investigate how different types of social support affect the relationship between Internet use and older adults' health status.

## Research purposes and hypotheses

**Figure 1:**
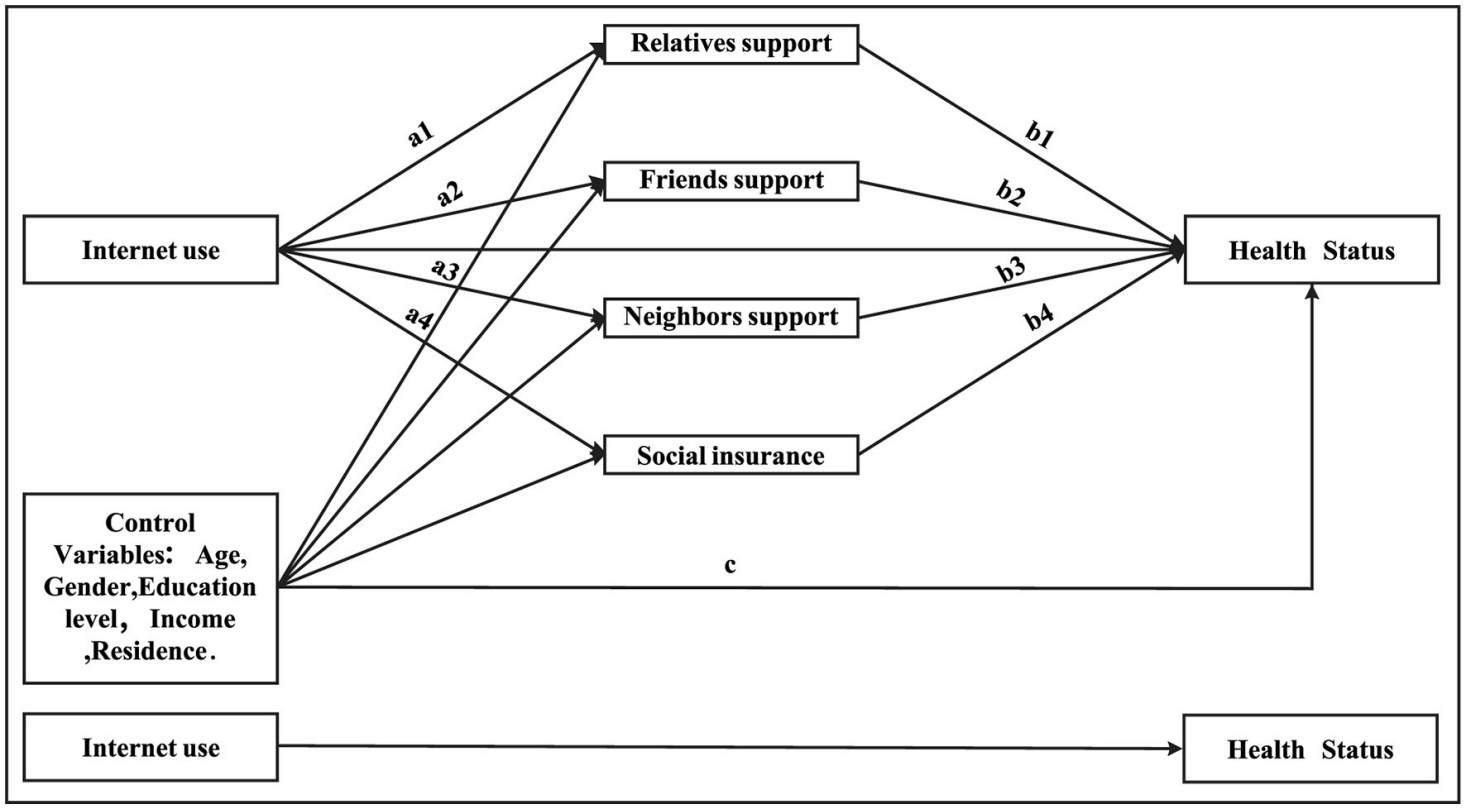
The hypothesized model.

The main purpose of this study is to explore the relationships among Internet use, social support, and health status among older adults in the Chinese context, and examine whether different forms of social support play mediating roles between Internet use and health. Therefore, the current study has the following three steps. First of all, the nationally representative CGSS survey data is used to comprehensively measure two types of social support (formal social support and informal social support) through four indicators: social insurance, relatives, friends and neighbors; Secondly, the multiple mediation analysis is applied to explore the path of Internet use influencing the health status of older adults in China through social support. Finally, on the basis of existing research, it is believed that social support has a positive impact on older adults' health status, which is helpful to provide policy references for improving older adults' health in China. The hypothesis proposed are as follows (Figure 1).

H1: Internet use has positive effects on the health status of older adults.
H2: Internet use has an indirect effect on the health status of older adults through relatives support.
H3: Internet use has an indirect effect on the health status of older adults through friends support.
H4: Internet use has an indirect effect on the health status of older adults through neighbors support.
H5: Internet use has an indirect effect on the health status of older adults through formal support.

## Methods

### Data sources

We use data from the 2021 Chinese General Social Survey (CGSS). This survey project mainly focuses on the major theoretical and practical issues in the changing social structure of China. It comprehensively collects some basic information on residents' behavior patterns, thinking patterns, lifestyles, and social changes. A total of 8,148 valid samples were completed in the 2021 CGSS. Currently, these data are suitable for this study because they collect rich information about Internet use, health, social support, and sociodemographic characteristics. Finally, a total of 2,929 older adults aged between the ages of 60 and 99 in urban and rural areas were included in this study.

## Measures

### Dependent variable

This paper analyzed the health status of older adults. According to the definition of health by WHO, health included “physical health, mental health, social adaptation, and moral health” (56), we used three questions to describe the health status. Consistent with the measurement of health in the study by Wang et al. (57), the question “What do you think of your current health?” was selected to measure the variable of self-rated health. Respondents' answers ranged from “very unhealthy” (=1), “relatively unhealthy” (=2), “average” (=3), “relatively healthy” (=4), and “very healthy” (=5). The question “In the past four weeks, how often has your work or other daily activities been affected because of the health issues?” was selected to measure the variable of physical health. The question “In the past four weeks, how often have your felt depressed?” For physical health and mental health, the rating regarding the frequency on a 5-point scale ranged from “always” (=1), “often” (=2), “sometimes” (=3), “rarely” (=4), and “never” (=5). We sum up the above questions and generate a new variable “health”. Considering the accuracy of the study, we use factor analysis to show the strength of the inter-correlation between the items and also test the reliability of the data through the Kaiser-Meyer-Olkin (KMO) index range from 0 to 1 and through the Cronbach's alpha. From Table 1, we can see that the KMO value is 0.67 and the Cronbach's alpha is 0.76, suggesting that the above measurements have high reliability.

**Table 1:** Factor analysis of items of health.

	**Variable**	**Uniqueness**	**Kmo**	**Kmo-overall**	**Cronbach α**
1	Subjective evaluation on their current physical health	0.493	0.654	0.667	0.762
2	The frequency of health problems affecting work or other daily activities over the past 4 weeks	0.427	0.627		
3	The frequency of depression over the past 4 weeks	0.651	0.760		

### Independent variable

The independent variable is Internet use. According to a study by Han et al. in 2021 (58), We chose the question “In the past year, what was your use of the internet (including mobile internet)?” to measure the use of the Internet in CGSS. Based on the respondents' answers, the rating in terms of the frequency on a 5-point scale ranged from “never”(=1), “rarely”(=2), “sometimes”(=3), “often”(=4) and “very frequently”(=5).

### Mediating variables

The mediating variable in this paper is social support. It could be divided into two aspects: informal social support and formal social support. As for informal social support, we apply Berkman and Cantor's conceptual framework (41), which describes informal social support along three key dimensions: relatives support, friends support and neighbors support. In CGSS, we use these questions in the questionnaire: “In the past year, have you often gathered with relatives who do not live together in your spare time?”, “How often do you socialize with your neighbors?”, “How often do you socialize with other friends?”, “In the past year, you gathered with friends in your spare time”. The rating in terms of frequency on a 5-point scale ranged from “never” (=1), “several times a year or less” (=2), “several times a month” (=3), “several times a week” (=4) and “every day” (=5). We sum the last two questions together and form a new variable “friends support”. All the respondents' answers to these questions reflect the informal social support of older adults.

Formal social support, as mentioned earlier, which divided into two parts: health insurance and endowment insurance in the Chinese context. In the CGSS questionnaire, respondents were asked to answer these two questions “Are you currently enrolled in any of the following social insurance programs? –Urban Employee Basic Medical Insurance (UEBMI), Urban Resident Basic Medical Insurance (URBMI), or New Rural Cooperative Medical Scheme (NCMS)” and we dichotomized their answers into “yes” = 1 and “no” = 0; “Are you currently enrolled in any of the following social insurance programs? –the rural endowment insurance or the urban endowment insurance” and we dichotomized their answers into “yes” = 1 and “no” = 0. We sum the above two together to form a new variable “social insurance” to measure formal social support.

### Control variables

Five variables are included: age, gender, education level, income, and residence. Gender is coded as 0 for females and 1 for males. Education is structured based on residents' educational attainment. It is categorized into primary education or below, primary school education, middle school education, high school education, and university education or above (education =0, 1, 2, 3, and 4). To correct for the positive skewness of income, we used the natural log transformation. The residence type is coded as 0 for rural areas and 1 for urban areas.

### Statistical analysis

Descriptive statistics and Pearson correlation analyses were performed using STATA17.0 software (two-sided test p < 0.05 was considered to be significantly correlated); We then performed an OLS regression controlling for gender, age, education, and income to examine the relationship between Internet use, social support, and health status. All control variables were assumed to have an impact on health. At last, in SPSS25.0, Model 4 in the process plugin compiled by Hayes (2017) was used for the multiple mediation effect analysis, and the bootstrap test was used to estimate direct effects and indirect effects according to repeated sampling from sample data, so as to establish confidence intervals for each effect. When the confidence interval does not contain 0, the corresponding effect is significant.

## Results

### Descriptive statistics and correlation analyses

Table 2 presents the descriptive statistics of key variables. The average age of the sample was 70.2 years old. About 51% were female.15.4% of the elderly have a senior high school education level. More than half of the participants came from rural areas (59.4%). After using the natural log transformation of income, the value range is 0 to 16.12. The average score of health was 10.4 (SD = 3.0). Notably, among the participants, more than half of the elderly have never used the Internet (61.4%). The mean score of friends support was 4.55 (SD =2.07). More than half of the participants (56.18%) showed that interactions with relatives were several times a year or less. However, 28.54% of the participants had never interacted with their neighbors. The average score of formal social support obtained by the sample was 1.74.

**Table 2:** Basic variable description statistics table.

**Variable**	**Mean/percentage**	**SD**	**Min**	**Max**	**Details**
Health	10.44	3.01	3	15	Continuous variables
Internet use	2.08	1.53	1	5	Multi-categorical variables
Never	61.43%				
Rarely	7.52%				
Sometimes	5.98%				
Often	11.51%				
Always	13.56%				
Relatives support	1.91	0.78	1	5	Multi-categorical variables
Never	28.83%				
Several times a year or less	56.18%				
Several times a month	10.78%				
Several times a week	3.25%				
Every day	0.96%				
Friends support	4.55	2.07	2	10	Continuous variables
Neighbors support	2.93	1.50	1	5	Multi-categorical variables
Never	28.54%				
Several times a year or less	9.55%				
Several times a month	24.42%				
Several times a week	15.73%				
Every day	21.77%				
Social insurance	1.74	0.52	0	2	Continuous variables
Income	8.34	4.41	0	16.12	Natural log transformation.
Gender	0.49	0.50	0	1	Binary variables
Female	51.31%				
Male	48.69%				
Education	1.50	1.12	0	4	Multi-categorical variables
Illiteracy	21.00%				
Primary school	32.70%				
Junior high school	26.32%				
senior high school	15.37%				
college or above	4.60%				
Residence	0.41	0.49	0	1	Binary variables
Rural	59.37%				
Urban	40.63%				
Age	2,929 (100.00)	70.23 (6.88)	60	99	Continuous variables

Table 3 presents the correlation matrix of the core studied variables. In general, positive correlations were found between the health status of older adults in China and Internet use, informal social support, formal social support, gender, age, residence, income, and education level, while age was negatively correlated with it. Internet use was significantly positively related to interaction with relatives and friends, formal social support, and other sociodemographic characteristics but negatively associated with age. There is a positive correlation between the three different types of informal social support. For formal social support, residence, income, and education level were all positively correlated with it.

**Table 3:** Pearson's correlations among relevant study variables.

**Variables**	**1**	**2**	**3**	**4**	**5**	**6**	**7**	**8**	**9**	**10**	**11**
1. Health	1										
2. Internet use	0.207^***^	1									
3. Relatives support	0.140^***^	0.143^***^	1								
4. Friends support	0.117^***^	0.142^***^	0.350^***^	1							
5. Neighbors support	0.0140	−0.0260	0.161^***^	0.493^***^	1						
6. Social insurance	0.052^***^	0.101^***^	0.038^**^	0.035^*^	−0.0100	1					
7. Gender	0.140^***^	0.0210	0.0190	0.0080	−0.083^***^	0.045^**^	1				
8. Age	−0.083^***^	−0.255^***^	−0.0140	−0.0100	0.0210	−0.055^***^	0.00500	1			
9. Residence	0.211^***^	0.314^***^	0.161^***^	0.110^***^	−0.092^***^	0.142^***^	0.0110	0.076^***^	1		
10. Income	0.134^***^	0.174^***^	0.072^***^	0.073^***^	−0.033^*^	0.097^***^	0.126^***^	0.00900	0.332^***^	1	
11. Education	0.243^***^	0.446^***^	0.134^***^	0.081^***^	−0.109^***^	0.144^***^	0.232^***^	−0.174^***^	0.451^***^	0.259^***^	1

^*^p < 0.05; ^**^ < 0.01; ^***^p < 0.001.

### Relationship between internet use and health

The main results showed that all nested regression models are statistically significant between models (Table 4). As the baseline model, Model 1 only examined the effects of control variables on health. We can obviously see that gender, age, education level, and income have a significant impact on the health of older adults. Model 2 analyzed the impact of Internet use on health. The results showed that the regression coefficient of Internet use to health is 0.2, and it is significant at the level of 0.001. This finding indicates that these two variables are highly correlated. That is, the more their use of the Internet, the higher level of their health. Model 3, Model 4, and Model 5 analyzed the three dimensions of informal social support, including the effects of relatives support, friends support, and neighbors support on health. The results showed that relatives support (β = 0.34, *p* < 0.001), and friends support (β = 0.09, *p* < 0.01) were positively associated with health, while neighbors support had no significant impact on health. Furthermore, the result of Model 6 showed that formal social support had no significant impact on health. In addition, regression analysis indicated that among the control variables, gender, age, education, residence, and income all passed the significance test in the six models, indicating that all five variables were significantly associated with health. For example, in Model 1, gender (β = 0.61, *p* < 0.001), education (β = 0.36, p < 0.001), residence (β = 0.87, *p* < 0.001), and income (β = 0.03, *p* < 0.05) were positively associated with health. In contrast, age was negatively associated with health (β = - 0.03, *p* < 0.01). Therefore, based on the regression coefficients for the three dimensions of informal support, relatives support had the largest impact on health, followed by support from friends. Neighbors support and formal social support had no significant correlation with health.

**Table 4:** Impacts of Internet use on Chinese older adults' health status: based on OLS model.

	**Model**	**Model**	**Model**	**Model**	**Model**	**Model**
	**1**	**2**	**3**	**4**	**5**	**6**
Gender	0.613^***^	0.649^***^	0.651^***^	0.669^***^	0.669^***^	0.667^***^
	(0.112)	(0.112)	(0.112)	(0.112)	(0.113)	(0.114)
Age	−0.033^***^	−0.024^**^	−0.024^**^	−0.025^**^	−0.025^**^	−0.024^**^
	(0.008)	(0.008)	(0.008)	(0.008)	(0.008)	(0.008)
Education	0.358^***^	0.266^***^	0.258^***^	0.257^***^	0.260^***^	0.260^***^
	(0.058)	(0.061)	(0.060)	(0.061)	(0.061)	(0.061)
Residence	0.871^***^	0.770^***^	0.695^***^	0.685^***^	0.687^***^	0.682^***^
	(0.130)	(0.131)	(0.131)	(0.132)	(0.133)	(0.134)
Income	0.030^*^	0.027^*^	0.026^*^	0.026^*^	0.027^*^	0.027^*^
	(0.013)	(0.013)	(0.013)	(0.013)	(0.013)	(0.013)
Internet use		0.200^***^	0.184^***^	0.175^***^	0.174^***^	0.167^***^
		(0.041)	(0.041)	(0.041)	(0.041)	(0.042)
Relatives support			0.345^***^	0.262^***^	0.262^***^	0.260^***^
			(0.070)	(0.075)	(0.075)	(0.075)
Friends support				0.088^**^	0.082^*^	0.082^*^
				(0.028)	(0.032)	(0.032)
Neighbors support					0.017	0.018
					(0.042)	(0.043)
Social insurance						0.004
						(0.107)
_Cons	11.307^***^	10.430^***^	9.877^***^	9.700^***^	9.686^***^	9.633^***^
	(0.594)	(0.618)	(0.626)	(0.632)	(0.636)	(0.668)
*N*	2,845	2,845	2,838	2,812	2,802	2,768
*R* ^2^	0.089	0.097	0.104	0.107	0.107	0.105

^*^p < 0.05; ^**^ < 0.01; ^***^p < 0.001.

### Mediation effect analysis

The results of the first and second parts in Table 5 showed that the coefficient of Internet use to social support were 1.634, 3.474,2.681, and 1.829, and a 95% confidence interval (CI) did not include 0. Meanwhile, the coefficient of social support to health were 0.260, 0.083, 0.018, and 0.004, and the 95% confidence interval (CI) did not include 0 among the two of them. Combining the results of Internet use on health, social support was considered to play a partial mediating role in the relationship between Internet use and health.

**Table 5:** Path-coefficients of the mediating models.

**Variables**	***B***	**BC95%LL**	**BC95%UL**	***R* ^2^**	***F***
**Internet use vs. informal social support**					
Internet use → relatives support	1.634	1.306	1.962	0.035	16.738
Internet use → friends support	3.474	2.596	4.352	0.027	12.619
Internet use → neighbors support	2.681	2.046	3.316	0.021	9.858
**Internet use vs. formal social support**					
Internet use → social insurance	1.829	1.612	2.046	0.033	15.685
**Social support vs. health**					
Relatives support → health	0.260	0.112	0.408		
Friends support → health	0.083	0.019	0.146		
Neighbors support → health	0.018	−0.066	0.102		
Social insurance → health	0.004	−0.207	0.214		
**Internet use vs. health**					
Internet use → health	0.167	0.085	0.248		

Model 4 of PROCESS was used to test the multiple mediating effects of social support. From the total effects in Table 6, Internet use had a significant positive effect on health [Bootstrap 95% CI:0.111, 0.274]. The direct effect results showed that Internet use had a significant positive effect on health [Bootstrap 95% CI:0.085, 0.248]. Thus, H1 was fully supported. Regarding the effects of social support on health status, the indirect effect results showed that among the three types of informal social support, two of them (relatives support and friends support) had significant and positive effects on the health of older adults in China ([Bootstrap 95% CI: 0.004, 0.022]; [Bootstrap 95% CI:0.002, 0.026]), whereas the other support had no significant effects. These findings fully supported H2 and H3, but H4 was not. With regards to formal social support, social insurance had no significant effect on this mechanism [Bootstrap 95% CI:−0.003, 0.003], and similarly, H5 was not verified. Among all indirect paths via social support, two mechanisms were significant: Internet use → relatives support → health; Internet use → friends support → health. Therefore, relatives support and friends support were mediators between Internet use and health. Moreover, the effect of formal social support on health did not have a mediation effect, but some informal support did. Thus, we can conclude that informal social support may play a more significant mediating role than formal social support.

**Table 6:** Mediating effects of social support.

**Paths**	**Standardized coef**.	**Bootstrap 95%CI**	
		**Lower**	**Upper**
**Total effect**			
Internet use → health	0.193	0.111	0.274
**Direct effects**			
Internet use → health	0.167	0.085	0.248
**Indirect effects (total)**	0.026	0.013	0.041
Internet use → relatives support → health	0.012	0.004	0.022
Internet use → friends support → health	0.014	0.002	0.026
Internet use → neighbors support → health	0.001	−0.004	0.006
Internet use → social insurance → health	0.001	−0.003	0.003
**Indirect effects contrast**			
Relatives support vs. Friends support	−0.002	−0.017	0.014
Relatives support vs. Neighbors support	0.011	0.002	0.022
Relatives support vs. Social insurance	0.012	0.004	0.023
Friends support vs. Neighbors support	0.013	−0.001	0.027
Friends support vs. Social insurance	0.014	0.003	0.026
Neighbors support vs. Social insurance	0.001	−0.004	0.006

### Difference between rural and urban areas in social support

We also used PROCESS to test for differences between rural areas and urban areas (Tables 7, 8). We found significant differences between rural areas and urban areas in social support. When social support was used as a mediating variable, relatives support was considered to play a partial mediating role in the relationship between Internet use and health status [Bootstrap 95% CI: 0.005, 0.040] in rural areas. However, this path was not significant in the urban sample. In urban areas, the results showed that friends support partially mediates the effect of Internet use on health status [Bootstrap 95% CI: 0.002, 0.034].

**Table 7:** Mediating effects of social support in rural areas.

**Paths**	**Standardized coef**.	**Bootstrap 95%CI**	
		**Lower**	**Upper**
**Total effect**			
Internet use → health	0.064	0.104	0.354
**Direct effects**			
Internet use → health	0.064	0.071	0.321
**Indirect effects (total)**	0.011	0.013	0.056
Internet use → relatives support → health	0.009	0.005	0.040
Internet Use → friends support → health	0.008	−0.005	0.028
Internet use → neighbors support → health	0.005	−0.005	0.014
Internet use → social insurance → health	0.002	−0.004	0.003

**Table 8:** Mediating effects of social support in urban areas.

**Paths**	**Standardized coef**.	**Bootstrap 95%CI**	
		**Lower**	**Upper**
**Total effect**			
Internet use → health	0.052	0.057	0.260
**Direct effects**			
Internet use → health	0.052	0.035	0.240
**Indirect effects (Total)**	0.009	0.005	0.041
Internet use → relatives support → health	0.005	−0.003	0.016
Internet use → friends support → health	0.008	0.002	0.034
Internet use → neighbors support → health	0.002	−0.006	0.004
Internet use → social insurance → health	0.004	−0.007	0.008

## Discussion

Using the 2021 wave of CGSS data, the present study examined the impact of Internet use on health status among Chinese older adults. Despite the large number of studies on the association between Internet use and health status, the potential mechanisms underlying this process remain to be explored. Therefore, we proposed a multiple mediation model to examine the role of social support. The results of this study show that Internet use was positively associated with health status by promoting social support. Several important conclusions can be drawn from this study.

The first finding is that Internet use has direct effects on health among Chinese older adults, which is in line with most prior work (59, 60). The mechanism of the effect of Internet use on the health status of older adults is that Internet use is conducive to expanding the scale of personal social networks, including communication with family and friends, access to effective information, and participation in leisure activities (61). On this basis, the Internet can provide individuals with more emotional comfort, alleviate negative emotions, provide them with more health information and medical resources, and improve their health (62), thus reducing their depression and loneliness and achieving higher life satisfaction (63). Our results are a good extension of previous studies that have simply separated Internet use into “use or not” (64, 65) to draw the general conclusion that Internet use has a positive effect on health status.

Second, this study distinguished three types of informal social support and examined the effect of each type on health separately. Our results show that not all types of informal social support have significant effects on the relationship between Internet use and health status, which is in line with prior studies. When compared to other types of informal social support, only relatives support and friends support significantly affected health status among Chinese older adults, which is consistent with previous research. For example, informal social support has been found to have a significant effect on health outcomes in a large number of studies, people with a higher level of informal social support have lower social pressure and higher health status (66, 67). For older adults, regular contact with relatives and friends can effectively promote health (68). Krause found that informal social support established through voluntary activities has a positive impact on the health of the elderly (69). Meanwhile, we found that relatives support and friends support significantly mediated the effect of Internet usage on health. This is consistent with previous research, suggesting that social support from relatives and friends partially mediated the relationship between Internet use and self-rated health. The reason may be that the Internet can increase the frequency of interaction between older adults and their relatives and friends, from which they can obtain social support (53). Social support means getting emotional encouragement, information, and help from relatives and friends they get along with. When older adults perceive higher levels of social support, they are more inclined to show better adjustment, thus resulting in better health. Therefore, the mediating role of relatives and friends support suggests that older adults can not only use the Internet to promote health but also use more relatives and friends support resources through the Internet to promote their health.

We also found that neighbors support did not have a significant influence on health status, which was contrary to our expectations. Prior research has empirically supported that neighborhood support can not only provide instant information, but also provide help at the first time in case of emergency. Therefore, neighborhood support helps to improve the mental health of older adults (70, 71). This inconsistent finding could be due to the difference in the Chinese situation. Unlike traditional society, rapid urbanization has brought about modern urban lifestyles and dramatic changes to traditional neighborhoods. In particular, the development of network technology has freed people from face-to-face and proximity forms of interaction, and people can reach the needs of social interaction without leaving their homes. Meanwhile, the diversification of modern life and leisure styles has greatly weakened the importance of neighborhood relations. Moreover, neighbors support did not significantly mediate the role between Internet use and self-rated health, this may be due to COVID lockdowns, people generally go out less and have less opportunity to contact their neighbors, which also affects the impact of neighbor support on health to some extent.

Third, our results show that the mediating pathway of Internet use on the health status of older adults through social insurance is not significant. That is, the underlying mechanism between Internet use and health status among older adults in China was informal social support only, which is contrary to our expectations. The critical role of social insurance in motivating health outcomes and promoting health equity has been empirically supported by a strand of previous research (72, 73). This inconsistent finding could be due to the difference in the measurement of social insurance, as well as whether to control the use of the Internet. Previous studies have generally focused on social insurance alone, so there is a need to consider the factors of Internet use to conduct more empirical studies and compare their effects on health status together.

The last and also most important finding in the current study is that regarding social support differences between urban and rural areas. For the rural elderly, relatives support has a significant mediating effect between Internet use and health status, while for the urban elderly, friends support has a significant mediating effect between Internet use and health status. The analysis is based on the following reasons: on the one hand, Chinese society is an “acquaintance society” characterized by relationship orientation (74, 75), and older adults have a higher consistency of family networks in rural areas of China. In line with past research, informal social support is also the main source of social support for rural residents (76). Compared with other social support, relatives of rural people are close. Relatives support can provide higher interpersonal trust and reciprocity, which will help to generate positive emotional experiences and improve the health status of older adults. On the other hand, with the reduction of urban family size and the diversity and convenience of urban social space, it is easier for older adults to establish ties with friends. When the elderly can no longer obtain continuous and sufficient family support, the support of friends' network, as the second source of social support in the social interaction of the elderly, has become more important for health (77, 78).

There are still some limitations in this article. First, due to the limitation of existing data, the Internet use of the elderly only measures the frequency of use, which cannot fully show the diversity of Internet use of the elderly, such as the time of use, psychological motivation, browsing items, etc. Second, in this paper, we analyze CGSS data only in 2021, without considering the survey data of other years, and cannot obtain the dynamic changes of the impact of social support on health status. Third, causal interpretation cannot only rely on path analysis, and future research should introduce longitudinal design or experimental research design. Last but not least, future studies should further explore the stories behind research findings through qualitative research methods such as interviews to enhance the richness of results.

## Conclusion

To sum up, this paper analyzed the influence mechanism of Internet use on the health status of older adults by focusing on the mediating role of social support. To be more specific, through the Internet, older adults in China can keep in touch with geographically dispersed relatives and friends, and establish new contacts with the outside world, which not only strengthens their connection with external social networks, but also increases communication with relatives, which is conducive to their health status. Overall, as there are many pathways for the Internet to affect health, social support only plays a partial mediating role in our study. That is, informal social support positively influenced the health status among older adults, while formal social support does not. These findings suggest that government should take effective steps to encourage and improve Internet use among older adults and obtain various social support to improve their health status.

## Data availability statement

The original contributions presented in the study are included in the article/supplementary material, further inquiries can be directed to the corresponding author.

## Author contributions

Conceptualization: YE and JY. Data curation, methodology, and writing—original draft: YE. Formal analysis: CL. Writing—review and editing: CL and LN. All authors have read and agreed to the published version of the manuscript.
